# Supplementary material for: Comparative evaluation of full-length isoform quantification from RNA-Seq
Source: BMC Bioinformatics. 2021 May 25;22:266. doi: 10.1186/s12859-021-04198-1 (PMC8145802; doi:10.1186/s12859-021-04198-1)
Supplement: Supplementary file 1 — Additional file 1. Supplemental Figures [file 12859_2021_4198_MOESM1_ESM.pdf]

Comparative evaluation of full-length isoform quantification from RNA-Seq

Dimitra Sarantopoulou[1,#a ¶], Thomas G. Brooks[1¶], Soumyashant Nayak[1], Anthonijo Mrcela[1], Nicholas F. Lahens[1], Gregory R. Grant[1,2*]

[1] Institute for Translational Medicine and Therapeutics, University of Pennsylvania, Philadelphia, Pennsylvania, United States of America

[2] Department of Genetics, University of Pennsylvania, Philadelphia, Pennsylvania, United States of America

[#a] Current address: National Institute on Aging, National Institutes of Health, Baltimore, Maryland, United States of America

[¶] equal contribution

Email: ggrant@pennmedicine.upenn.edu (GG)

Supplemental Figures

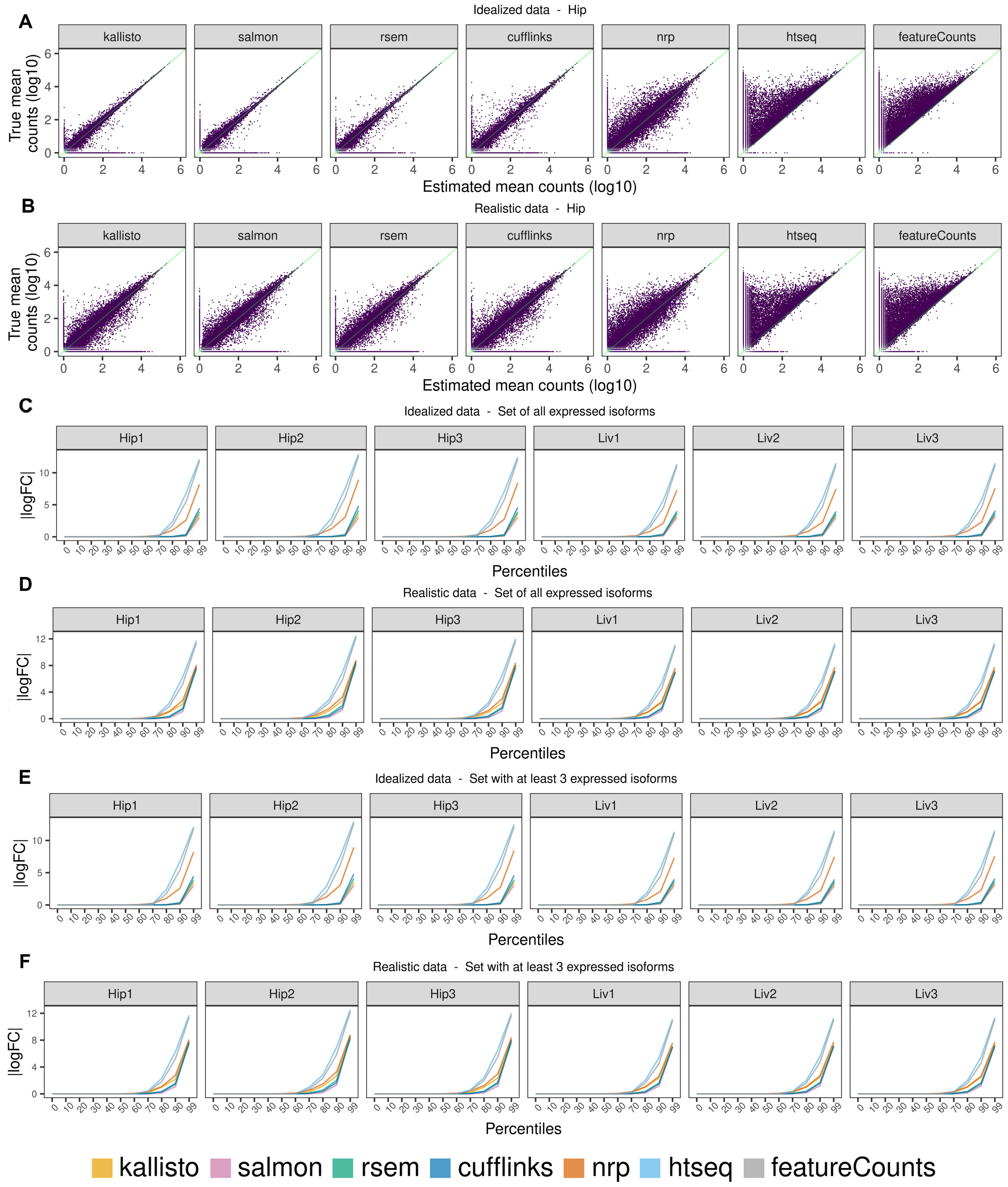

S1 Fig. Method effect on full-length isoform quantification using simulated data.

Method effect on full-length isoform quantification using simulated data. Average expression of three hippocampus samples, comparing each method to the truth, using A) idealized and B) realistic data. Percentiles of cumulative distribution of |logFC| using C) idealized data, D) realistic data, E-F) idealized and realistic data respectively, where we restricted to the set of genes that have at least 3 expressed isoforms.

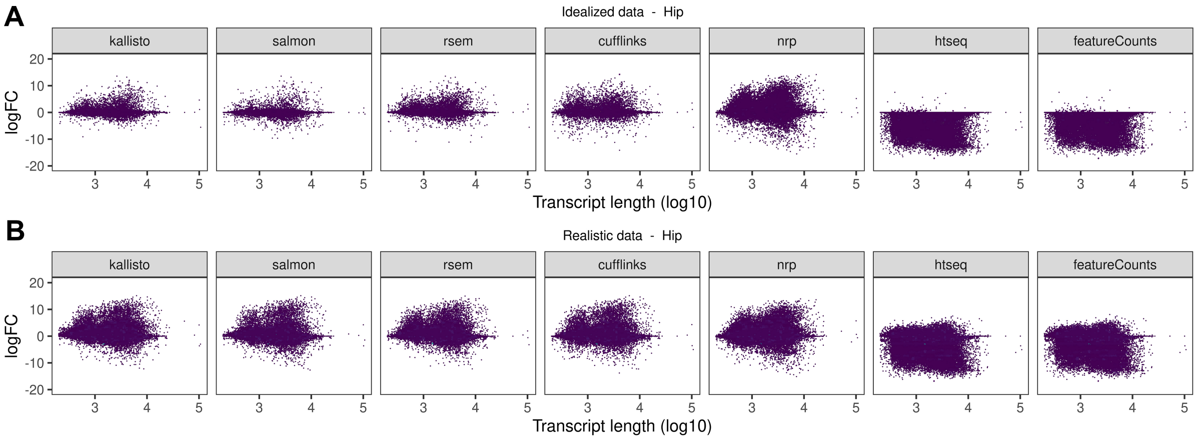

S2 Fig. Effect of transcript length on quantification accuracy.

Effect of transcript length on quantification accuracy, given by adjusted logFC of the average of the three hippocampus samples, using A) idealized and B) realistic data.

fig

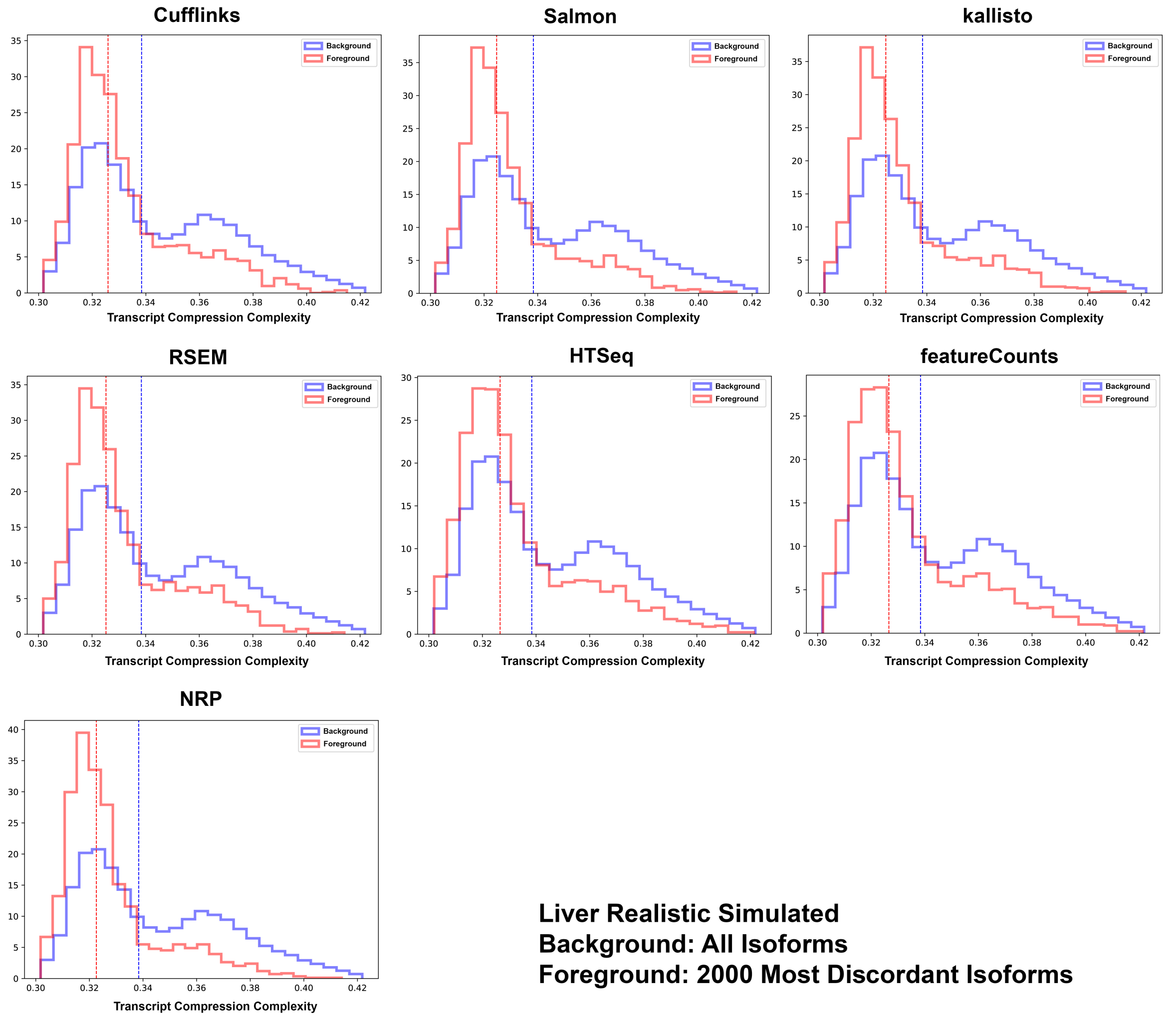

S3 Fig. Differential distribution of transcript compression complexity.

For each method the foreground and background distributions are shown for transcript compression complexity. The background is over all isoforms, the foreground is over the top 2,000 discordant transcripts sorted by absolute logFC. The foreground distribution is highly enriched for low compression complexity for all methods.

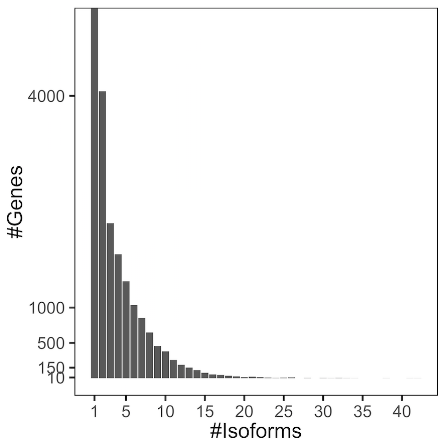

S4 Fig. The distribution of the #genes according to the #annotated isoforms.

The distribution of the number of genes for different number of annotated isoforms.
